# Complications in post-mortem GHB cut-off values in urine samples: A case report

**DOI:** 10.1016/j.toxrep.2023.05.002

**Published:** 2023-05-09

**Authors:** N.H.C. Loos, K.E. van den Hondel, U.J.L. Reijnders, E.J.F. Franssen

**Affiliations:** a The Netherlands Cancer Institute, Division of Pharmacology, Amsterdam, the Netherlands; b OLVG Hospital Amsterdam, Department of Hospital Pharmacy, Amsterdam, the Netherlands; c Public Health Service, Department of Forensic Medicine, Amsterdam, the Netherlands

**Keywords:** Gamma-hydroxybutyric acid (GHB), Urine cut-off reference values, Post-mortem, Forensic toxicology

## Abstract

Gamma-hydroxybutyric acid (GHB) is a drug of abuse, that interplays with a GABAergic system, resulting in an euphoric state and increased mood and impulses. Two cases of fatal mixed intoxications including GHB intake are presented here. In both cases, GHB was used together with multiple other drugs. Interpretation of GHB cut-off values are complicated in post-mortem analysis, because GHB can be post-mortem formed. The post-mortem GHB formation is dependent of the post-mortem interval (PMI) and the storage conditions of the samples. The GHB concentrations in urine are more stable compared to blood samples, when the samples are stored at the correct way at − 20 °C. Therefore, urine is the recommended matrix to analyze in toxicological screenings, since it allows more specific determination of exposure to exogenous GHB. Different cut-off values are used for matrices from living and deceased people. A cut-off value of 30 mg/L is recommended to discriminate between endogenous concentrations and concentrations resulting from exogenous GHB exposure. Moreover, post-mortem GHB formation can take place before sampling. However, when the samples are immediately stored at cooled conditions, no in vitro formation of GHB will take place. Urinary screening of GHB may serve as an initial screening for estimation of exposure of GHB in the body. However, additional quantitative GHB analysis in blood is required to estimate GHB exposure at the time of death. Furthermore, to obtain more reliable results for the ante-mortem GHB exposure, it may be useful to measure other biomarkers, like some GHB metabolites, especially in blood.

## Highlights

• Two cases of fatal mixed intoxications including GHB are presented here.
• Interpretation of GHB cut-off values are complicated by post-mortem GHB formation.
• Urinary toxicology screening allow an estimation of GHB exposure at time of death.
• Immediate sample storage at cooled conditions will prevent post-mortem GHB formation.
• Biomarkers as GHB metabolites, might give more reliable insights in the GHB intake.

## Graphical Abstract

**Figure:**
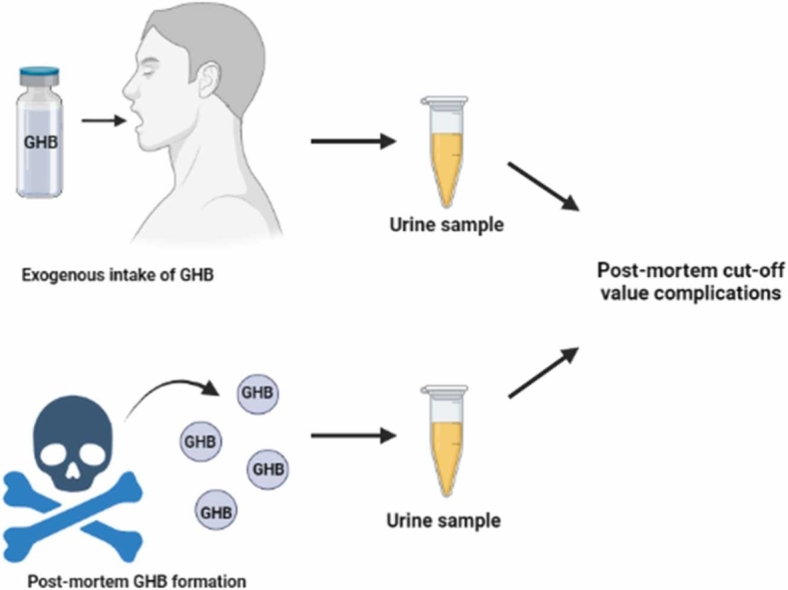

## 1 Introduction

Gamma-hydroxybutyric acid (GHB) is an endogenous compound acting at the central nervous system [1]. GHB is a metabolite of the neurotransmitter Gamma Amino Butyric Acid (GABA) and is present in the central nervous system and peripheral tissues [2]. It is an illicit drug, used for recreational purposes and is often used in sexual assaults (so called ‘date-rape drug’) [1]. The pharmacological effects of GHB are dose dependent. Doses up to 1.5 g result in an euphoric state and lead to an anxiolytic effect, while doses up to 2.5 g increase the mood, impulse and sexual activity. Intake of even higher doses will lead to a sudden deep sleep [3]. However, there are also therapeutic registered formulations of GHB, including its sodium salt (Xyrem®), which is indicated for narcolepsy-associated cataplexy, and (Alcover®), which is used as adjuvant for detoxification and withdrawal in alcoholics [1]. GHB could interplay in the GABAergic system, especially through binding to the GABA_B_ receptor, in which it fulfills the role of neurotransmitter or neuromodulator [1]. Precursors of GHB like gamma-butyrolactone (GBL) and 1,4-butanediol (1,4-BD), which are not regulated, are rapidly converted into GHB (t_1/2_ ≈ 1 min) [1], [4]. The half-life of GHB itself is around 30–50 min, and therefore GHB breakdown will go relatively fast in a living person, which could also be a limiting factor for post-mortem analysis when this person died [1]. Only 1–5 % of the used dose is recovered in urine and the time window for quantification is relatively short (3–10 h) [1]. Clinically relevant GHB intoxications are characterized by bradycardia, seizures, respiratory depression, and loss of consciousness and coma [5], [6], [7], [8]. Fatal overdoses are described for GHB itself, but also for its precursors GBL and 1,4-BD [9], [10], [11]. The lethal plasma concentration of GHB is approximately 500 mg/L, leading to cardiorespiratory depression [1]. Urinary GHB screening may routinely be included in initial comprehensive forensic toxicology screening to estimate potential ante-mortem GHB exposure in post mortem cases. However, in cases of positive tests (GHB in urine > 30 mg/L) additional GHB quantification in post-mortem blood should be performed [12].

## 2 Cases of fatal mixed intoxications including GHB

In this case report, we will discuss two cases in which the deceased person had used GHB amongst other drugs. When there is a suspicion of an unnatural cause of death in the Netherlands, a forensic physician will perform an external examination at the scene and femoral blood and urine samples are collected from the body [13]. However, after a toxicological screening with a quick test (Drugs of Abuse-immunoassay), there was an indication for a mixed drug intoxication in potential lethal doses. There were no indications for a criminal offense and therefore forensic autopsies were not indicated in both cases. The first case concerns a man, who was found surrounded by different drugs. He met two other persons the night before and they went to his place to take drugs. The man was found the next morning and his death was confirmed by authorities. At least remnants of crystal meth, MDMA, cannabis and GBL (gamma-butyrolactone, a GHB analog) were found in the house. Moreover, a basepipe, injection needles, clear fluids and medication strips (of venlafaxine, methylphenidate, somatropin and propranolol) were discovered. At the external examination, nothing particular was noted and a rectal body temperature of 34.4 °C was measured 4.5 h at the confirmation of death by authorities. At the time of temperature measurement of the naked and dry body, the ambient temperature was 19.6 °C with stationary air. The post-mortem interval (PMI), so the time that is elapsed since the time of death, based on the nomogram of Henssge [14], was estimated to be approximately 6 h. Due to the fact that the PMI was several hours, the estimation of body temperature is not reliable, and could therefore be lower at the time of death. Moreover, post-mortem blood analysis showed the presence of amphetamine, methamphetamine, quinine, and venlafaxine (including its metabolite). GHB, amphetamines, cannabis and MDMA were detected in the urine sample. A concentration of 35 mg/L of GHB was measured in urine, in blood the GHB concentration was < 10 mg/L.

The second case regards a male, who was found dead in his residence. The night before he went to a party, where he used MDMA and GHB. The morning after, he felt sick, including vomiting, diarrhea and pain in his feet. He took two capsules of 50 mg tramadol and he was feeling hot. A couple hours later, he was found dead in his bed surrounded by lots of vomit. At the external examination, nothing specific was noticed and the body temperature was 33 °C at a PMI of two hours. At the time of temperature measurement of the naked and dry body, the ambient temperature was 19 °C with stationary air. The forensic physician had a suspicion of drug abuse, so blood and urine samples were collected at the scene and analyzed. Initial comprehensive toxicological screening in blood revealed the presence of several drugs, including cocaine, benzoylecgonine, ecgonine methyl ester, cocaethylene, MDMA, diazepam, alprazolam, quetiapine, norquetiapine and paracetamol. In addition, the urine sample showed a variety of drugs of abuse, such as GHB (72 mg/L), amphetamines, benzodiazepines, cannabis, cocaine and MDMA. The GHB concentration in blood was < 10 mg/L.

In both cases, there was a mixed intoxication, which could result in some analytical problems during the substance analysis, such as cross-reactivity. Alcohol was also determined in urine and blood, which may interfere when an enzymatic assay of GHB was used [15], [16]. In the second case, the alcohol level in blood was 1.6 g/L and 2.3 g/L in urine.

## 3 Methods of GHB and other drug of abuse determination

GHB concentration were measured with a validated gas chromatography with flame ionization detection (GC–FID) system. This GC-FID system consisted of an HP 6890 Series G1530A Gas Chromatograph, a G2613A/G2614A automatic liquid sampler, and a microcell electron capture detector. A Chrompack, WCOT-fused silica capillary column (CP-SIL 5 CB, 25 m·530 µm, film thickness 500 µm; Agilent Technologies) column was used at a temperature of 120 °C and with у-Valerolactone as internal standard [15]. This method was based on the GHB conversion to gamma-butyrolactone in an acid environment [17]. Although this method is time consuming, because sample pretreatment including obtaining the results takes around 90 min, this is the method of choice to measure GHB concentrations [15]. More recently, more rapid enzymatic assays became available, but their reliability was inconclusive and these methods are susceptible for cross-reactivity with other small compounds, such as ethanol and vitamin C [15], [16]. In the second case, there was alcohol intake, but this did not interfere with the bioanalytical measurement of GHB by the chromatographic assay used for the GHB quantification. In addition, volatile substances in blood and urine were determined with gas chromatography; Drugs of abuse (cocaine, amphetamines, cannabis, etc.) were determined with immunochemistry techniques (Beckman, EMIT), drugs in blood were determined with liquid chromatography equipped with mass spectrometry detection (LC-MS^n^, ToxTyper™).

## 4 Complications of interpretation of urine cut-off values

One of the major challenges in post-mortem GHB analysis is the cut-off value in the analyzed matrices. The cut-off value is a predefined value to which results will be reported as positive, when the measured value is equal to or above this cut-off value. Furthermore, cut-off values are bioanalytical method dependent as immunoassays are more susceptible for cross reactivity compared to methods based on chromatography or mass spectrometry. However, in post-mortem GHB analysis, the interpretation of cut-off values is not straightforward, which is caused by the range of endogenous GHB concentrations and the possibility of post-mortem GHB formation. This complicates the interpretation of the measured values even further due to the increased GHB levels in blood as well as urine over time [8]. Therefore, the cut-off value for post-mortem GHB analysis is a higher value then the concentration levels, which can be explained by endogenous formation including post-mortem GHB formation. The normal post-mortem GHB formation is based on the sum of the interval between death and the sample collection and the period of transport and storage of the samples. However, GHB is post-mortem more stable in urine than in blood, and post-mortem formation of GHB in the bladder is less of a problem compared to post-mortem blood samples [18], [19], [20]. Therefore, there is more focus on the analysis of urine samples as body fluid for toxicological analysis. The PMI could also strongly affect the post-mortem GHB formation in blood and urine samples, which could be especially relevant for the first case due to the longer PMI. Although if the storage conditions of the samples were correct, so frozen at − 20 °C after sampling, there will be less post-mortem GHB formation in the samples [21]. It is worth noting, that GHB could be formed in the period between the time of death and the sampling.

Therefore, recommendations of cut-off values are different for specimens of living and deceased people [8]. The urine GHB cut-off levels for ante-mortem sampling are between 2 and 10 mg/L, whereas a cut-off of 6 mg/L is recommended [8], [22], [23], [24]. However, other studies are reporting 10 mg/L as ante-mortem cut-off value for urine [3], [8]. This cut-off value for ante-mortem GHB sampling could even lead to false negatives as shown by Mari et al.*,* because discrimination between endogenous formation and exogenous intake of GHB is difficult at this cut-off level [25]. Therefore, these authors recommend to use a cut-off value between 3 and 10 mg/L as indication for exogenous GHB intake [25].

In the study of Andresen et al., they analyzed the GHB concentrations in different specimens of 64 autopsy cases. They concluded that the GHB levels were the highest in heart blood, followed by venous blood. On average, urine GHB concentrations were low compared to blood levels, however the urine samples with high GHB exposure were in the same range as their corresponding blood samples [8]. Furthermore, based on this study, an interpretative cut-off value for urine should be 30 mg/L to discriminate between endogenous and exogenous GHB. In this study, they showed that 24 % of the cases exceeded GHB levels above 10 mg/L, and 6 % of the cases reached 20 mg/L. Therefore, a cut-off value of 30 mg/L is recommended based on these results [8]. The recommendation is only applicable when the samples are analyzed on the same day or directly stored at − 20° [8]. The importance of the storage conditions of the samples has also been implicated by the study of Kietzerow et al. [26]. They showed that the storage conditions of the specimens until analysis were the main cause of high artificial post-mortem GHB concentrations [26]. Furthermore, they stated that this increase in GHB levels in urine samples will only occur when samples are not immediately stored under cooled conditions [26]. The changes in GHB concentrations seems to be affected during the PMI in the deceased body, and the storage conditions after sampling [26]. Interestingly, this also seems to be subject to inter-individual variability [26].

For the identification of exogenous GHB intake in post-mortem blood samples, a cut-off value of 20–30 mg/L is accepted for venous blood and 50 mg/L for cardiac blood [12]. For ante-mortem samples, the cut-off value of 4 mg/L is significantly lower for serum samples [8], [24]. In our cases, the initial toxicology screen in urine revealed potential exogenous GHB exposure (cut-off > 30 mg/L). In contrast, additional GHB analysis in post mortem femoral blood did not reveal relevant GHB levels (< 10 mg/L). Furthermore, for a more reliable determination of GHB intake in post-mortem toxicology investigation in blood, it may be helpful to measure other biomarkers, like alpha and beta metabolites of GHB, such as 2,4-dihydroxybutyric acid (2,4-OH-BA), 3,4-dihydroxybutyric acid (3,4-OH-BA) or glycolic acid (GA) [3], [27], [28]. Analysis of 2,4-OH-BA, 3,4-OH-BA and GA in post-mortem blood samples may result in an additional benefit according to the study of Jarsiah et al. [3]. GA concentrations were higher in 6 of 11 femoral blood samples, and in 7 of 10 urine post-mortem samples of deceased people due to GHB intoxications compared to samples without exposure to GHB [3]. In all 11 measured samples of femoral blood of GHB intoxicated men, the levels of 2,4-OH-BA and 3,4-OH-BA were above the highest concentration in the non-GHB users [3]. Urinary concentrations seems to be not that valuable as femoral blood concentrations of these metabolites according to the authors [3]. They stated that femoral blood concentrations of > 4 mg/L for 2,4-OH-BA, > 5 mg/L for 3,4-OH-BA and > 12 mg/L for GA give indications for a lethal GHB intake [3]. Although, in the cases discussed here, there were no lethal GHB concentrations measured, the measurement of these metabolites could be considered in the future to further optimize the post-mortem GHB toxicological analysis.

## 5 Conclusion

In conclusion, the method of choice for GHB in post-mortal blood and urine is GLC. Furthermore, the use of a reliable cut-off value is very important to discriminate between endogenous concentrations and concentrations resulting from exogenous GHB exposure. Based on larger analytical studies, it should be recommended to use a cut-off value of 30 mg/L for the urine matrix in post-mortem analysis. This means that both cases discussed here, have GHB urine concentrations above this cut-off value. However, the measured concentrations in blood were below the serum cut-off value of 20–30 mg/L for both cases. Urinary detection of GHB may serve as a preliminary pre-screening for the exposition of GHB in the body at the time of death. However, additional GHB analysis in post mortem blood is required to estimate exogenous GHB exposure at the time of death.

## CRediT authorship contribution statement

**N.H.C. Loos:** Conceptualization, Visualization, Writing – original draft. **K.E. van den Hondel:** Sample collection, Writing – review & editing. **U.J.L. Reijnders:** Supervision, Writing – review & editing. **E.J.F. Franssen:** Sample analysis, Conceptualization, Supervision, Writing – review & editing. All authors commented on and approved the manuscript.

## Funding sources

This research did not receive any specific grant from funding agencies in the public, commercial, or not-for-profit sectors.

## Declaration of Competing Interest

The authors declare that they have no known competing financial interests or personal relationships that could have appeared to influence the work reported in this paper.

## Data Availability

The data that has been used is confidential.
